# Short-term traffic speed prediction under different data collection time intervals using a SARIMA-SDGM hybrid prediction model

**DOI:** 10.1371/journal.pone.0218626

**Published:** 2019-06-26

**Authors:** Zhanguo Song, Yanyong Guo, Yao Wu, Jing Ma

**Affiliations:** 1 Jiangsu Key Laboratory of Urban ITS, Southeast University, Nanjing, Jiangsu, China; 2 Jiangsu Province Collaborative Innovation Center of Modern Urban Traffic Technologies, Southeast University, Nanjing, Jiangsu, China; 3 Intelligent Transportation System Research Center, Southeast University, Nanjing, Jiangsu, China; 4 School of Transportation, Southeast University, Nanjing, Jiangsu, China; 5 Periodical Office, Chang’an University, Xi’an, Shaanxi, China; Central South University, CHINA

## Abstract

Short-term traffic speed prediction is a key component of proactive traffic control in the intelligent transportation systems. The objective of this study is to investigate the short-term traffic speed prediction under different data collection time intervals. Traffic speed data was collected from an urban freeway in Edmonton, Canada. A seasonal autoregressive integrated moving average plus seasonal discrete grey model structure (SARIMA-SDGM) was proposed to perform the traffic speed prediction. The model performance of SARIMA-SDGM model was compared with that of the seasonal autoregressive integrated moving average (SARIMA) model, seasonal discrete grey model (SDGM), artificial neural network (ANN) model, and support vector regression (SVR) model. The results showed that SARIMA-SDGM model performs best with the lowest mean absolute error (MAE), mean absolute percentage error (MAPE), and the root mean square error (RMSE). The traffic speed prediction accuracy under different time intervals were compared based on the SARIMA-SDGM model. The results showed that the prediction accuracy improves with the increase in time interval. In addition, when the time interval is greater than 10 min, the prediction results yield stable prediction accuracy.

## Introduction

There has been an increasing growth in traffic demand over the past two decades around the world. Transportation engineers are being challenged by the ever-increasing traffic demand and the corresponding traffic congestion and safety issues [1–4]. Many solutions have been investigated to mitigate the traffic congestion, in which the proactive traffic control system is great importance and efficient [5]. Specifically, short-term traffic prediction is an important component of proactive traffic control system. Traffic parameters including traffic flow, occupancy and traffic speed are the dominate variables in short-term traffic prediction.

Although each of the three traffic parameters can be used to describe traffic congestion, both traffic flow and speed have correlated with occupancy [6]. Compared to the traffic flow, one speed is mapped to one occupancy, whereas one traffic flow can be mapped to two occupancies [7–10]. In addition, speed is more directly related to the traffic operation statues. Besides, the real-time dynamic traffic guidance control strategy relies on the short-term traffic speed prediction results. As such, short-term traffic speed prediction has been identified as a key task for developing proactive traffic control system.

The specification of time intervals for data collection is a fundamental determinant of the nature and utility of traffic condition data. In the process of short-term traffic prediction, data collection time interval serves as the aggregation interval of traffic speed [11]. The data collection time interval provides the forecasting horizon for one-step-ahead forecasting. The accuracy of traffic prediction results highly lay on the data collection time interval. Nevertheless, the need for more rigorous understanding of the effects of data collection time interval specification within the context of short-term traffic condition forecasting is not well recognized. By contrast, it has been common practice in previous research to arbitrarily select the data collection time interval without consideration of time interval effects on the prediction results. Moreover, understanding the impact of data collection time interval on short-term traffic prediction can provide insights into the performance of prediction results. Moreover, different applications require different data collection time intervals. For example, predictive route guidance application requires a longer time interval, whereas traffic flow rate prediction needs a shorter time interval [12]. The data collection time interval is particularly important to the traffic speed prediction. The traffic speed prediction with a large time interval has limited capacity to reflect the dynamic traffic operation status. Thus, the prediction results are unable to be applied in traffic control strategy. Whereas, if the time interval is too small, the calculation is time consuming and the traffic speed prediction results are unstable. In addition, the collection process will result in missing information when the time interval is too small. As such, it is necessary to investigate the data collection time interval for short-term traffic prediction, especially for the traffic speed prediction where the speed data is discrete across time intervals.

The objective of this study is to investigate the short-term traffic speed prediction under different data collected time interval. Specifically, a seasonal autoregressive integrated moving average plus seasonal discrete grey model structure (SARIMA-SDGM) was proposed in this study. Speed data with various time intervals collected from an urban freeway in Edmonton, Canada were used. For model comparison purpose, four candidate methods, including seasonal autoregressive integrated moving average (SARIMA) model, seasonal discrete grey model structure (SDGM) model, artificial neural network (ANN) model, and support vector regression (SVR) model, were estimated and compared with SARIMA-SDGM model. Three indicators including the mean absolute error (MAE), mean absolute percentage error (MAPE), and the root mean square error (RMSE) were used to measure the models’ performance as well as the impact of time interval on traffic speed prediction accuracy. The main contributions of the study are: (a) this paper investigate the short-term traffic speed prediction under different data collection time intervals; and (b) a SARIMA-SDGM hybrid prediction model was proposed this paper and compared to the traditional methods (i.e. SARIMA and SDGM) and machine learning methods (i.e. ANN model and SVR model).

## Literature review

### Short-term traffic condition prediction methods

The past decades has seen a growth in the short-term traffic condition prediction studies. Various approaches have been applied in traffic condition forecasting. Traditionally, the parametric and nonparametric methods are two main methods that are used in short-term traffic condition predictions. A method can be thought paramedic when structure is fixed and parameters are learned from data set [13]. Likewise, nonparametric methods derive dynamic relationships directly from observed data and therefore are usually called data-driven approach.

The typical parametric methods are the autoregressive integrated moving average (ARIMA) model [14–16], and its extended structures, such as Kohonen-ARIMA model [17], seasonal autoregressive integrated moving average (SARIMA) model [18], and ARIMA with Kalman filter [19]. Other commonly used parametric methods include time series models [20] and spectral analysis [21–22]. The parametric methods are easy to be implemented and provide explicit theoretical interpretability with clear calculation construction. However, the parametric methods require high quality of data set. The traffic data sequence should be accurate and stable, which against the fact that the traffic data are stochastic and unstable. Therefore, these models are difficult to obtain accurate prediction results from the actual traffic data.

Comparing to the parametric methods, nonparametric methods derives the prediction results directly from data training. Due to the learning ability and strong generalization, the nonparametric methods are able to achieve better prediction accuracy. Numerous methods are used as the nonparametric methods including, the k-nearest neighbor approach [23–24], multi-type neural network [25–26], artificial neural network (ANN) model [27], kernel smoothing [28], and support vector regression (SVR) model [29]. Nonparametric methods enable the adaptive learning of potential traffic dynamics through historical traffic data, and have the desirable attribute of adapting to changing traffic condition. However, concerns with these methods are black box framework, difficult in model training. Besides, expanding the database needed for the adaptation decreases the computational efficiency.

Considering that each prediction method has its own application and advantage, recent studies have utilized the hybrid methods combining merits of different methods in short-term traffic condition prediction to improve the prediction accuracy. These methods include hybrid fuzzy rule-based approach [30], Bayesian-neural network approach [31], and chaos-wavelet analysis-support vector machine approach [32]. Generally, the hybrid prediction model can achieve better results than single prediction model. Moreover, the hybrid models are verified with higher prediction accuracy [33–34].

### Short-term traffic speed prediction

Numerous studies have investigated the short-term traffic speed prediction which is a kind of time series prediction [35]. Linear time series models have been widely used, including ARIMA model [14–15, 36], the seasonal ARIMA (SARIMA) model [37], and the exponential smoothing model [38]. However, the above-mentioned linear time series models require accurate and stable traffic speed data, whereas the actual traffic speed data are nonlinear and unstable. Therefore, these models cannot implement accurate forecast for traffic speed data that have nonlinear structure.

In recent years, with the development of machine learning technology, various machine-learning models have been adopted in traffic speed prediction. These models include support vector regression (SVR) [29], long short-term memory networks (LSTM) [39–40], and evolving fuzzy neural network (EFNN) [33]. Wang et al. [41] proposed a bidirectional long short-term memory neural network (Bi-LSTM NN) model in traffic speed prediction. The results showed that the proposed model outperforms ANN model. Ma et al. [42] utilized a convolutional neural network (CNN) to predict network-wide traffic speed. The results showed that the proposed method outperformed LSTM model by a mean squared errors improvement of 42.91%. Using the traffic speed data from the Caltrans Performance Measurement System (PeMS), Liu et al. [43] predicted traffic speed by the attention convolutional neural network (ACNN) model and found that the proposed model achieved better forecast results than traditional linear models.

In addition to the time series features, traffic speed is also influenced by geographical location and spatial correlation. Thus, the prediction models which consider the spatial features were proposed. These models include vector autoregressive (VAR) model [44], statistical analysis model (SAM) [45], the grey prediction model with Fourier error correction (EFGM) [46], and the grey prediction model with Markov chain (MKGM) [47]. In these models, the prediction results were achieved by exploring the road network and capturing the correlation information of the network.

Hybrid models were also applied in short-term traffic speed prediction. The temporal-spatial hybrid model was proposed to provide a complete description of the temporal-spatial interaction [48]. The spatial-temporal random effects (STRE) model was applied in traffic speed prediction by considering the spatial-temporal features of traffic speed [49]. The deep learning method combined with median filter preprocessing model (DLM8L) uses convolutional neural network (CNN) to extract temporal-spatial features and forecast traffic speed in highway [50]. Intuitively, the hybrid models can achieve better prediction results than single models [33–34, 37]. However, the estimation of the hybrid models is complex and require more effort, thereby discouraging the wide-scale implementation [21].

The literature review showed that most of short-term traffic speed predictions are based on time series models, spatial correlation models, and hybrid models. Compared to a single short-term traffic speed prediction model, a hybrid model can provide complex interpretability but achieve better accurate results. Few studies investigated the data collection time interval in short-term traffic prediction. However, different data collection time interval may have impact on the traffic speed prediction results.

## Methodology

This study proposes a hybrid prediction model framework by combining the SARIMA model with SDGM model to deal with traffic speed based on temporal and spatial seasonal characteristics.

### SARIMA model

SARIMA model is a commonly used time-series prediction method proposed by Box et al. [51]. As an improved form of ARIMA model, SARIMA model is used for periodic time series and performs the seasonal difference based on the ARIMA model. In addition, SARIMA model has been shown to effectively capture the seasonal feature of the time series, especially in the traffic speed time series [33, 34, 37, 52].

Based on the ARIMA(*p*, *d*, *q*) model which includes autoregressive (AR) algorithm and moving average (MA) algorithm, the SARIMA (*p*, *d*, *q*)(*P*, *D*, *Q*) model can be defined in (1). In this study, the SARIMA model is used to remove the autocorrelation structure from the time series so as to generate the residual series for the statistical tests in the heteroscedasticity test.

$$(B)\Phi (B^{S}){\left(1-B^{S}\right)}^{D}{\left(1-B\right)}^{d}X_{t}\;=\;\theta (B)\Theta (B^{S})\varepsilon_{t}$$ (1)

where *t* is time index; *ε*_*t*_ is the residual series; *p* is order of the short-term AR polynomial; *q* is order of the short-term MA polynomial; *d* is order of the short-term differencing; *P* is order of the seasonal AR polynomial; *Q* is order of the seasonal MA polynomial; *D* is order of the seasonal differencing, *B* is backshift operator such that *BX*_*t*_ = *X*_*t*−1_ = *ε*_*t*_ = *random error at time t*; (1 − *B*^*S*^)^*D*^ is seasonal differencing; (1 − *B*)^*d*^ is short-term differencing; *ϕ*(*B*) = 1 − *ϕ*_1_(*B*) − *ϕ*_2_(*B*)^2^ − ⋯ − *ϕ*_*p*_(*B*)^*p*^ is short-term AR polynomial; *θ*(*B*) = 1 − *θ*_1_*B* − *θ*_2_*B*^2^ − ⋯ − *θ*_*q*_*B*^*q*^ is short-term MA polynomial; Φ(*B*^*S*^) = 1 − Φ_1_(*B*^*S*^) − Φ_2_(*B*^*S*^)^2^ − ⋯ − Φ_*p*_(*B*^*S*^)^*p*^ is seasonal AR polynomial; and Θ(*B*^*S*^) = 1 − Θ_1_(*B*^*S*^) − Θ_2_(*B*^*S*^)^2^ − ⋯ − Θ_*Q*_(*B*^*S*^)^*Q*^ is seasonal MA polynomial.

For the processing of SARIMA model, three steps are used in the Box-Jenkins framework, i.e., model identification, model estimation, and model prediction [51]. In the model identification step, the periodic features of time series are identified. The periodic features are regarded as the criteria for applying the model [33–34, 53]. In the model estimation step, the model parameters are estimated using the maximum likelihood approach or least squares approach. In the model prediction step, forecast was obtained by the estimated model. In this study, these three steps are implemented using the SAS PROC [54]. The SARIMA algorithm in SAS is shown in the Algorithm 1.

**Algorithm 1: SARIMA**

**Input:** measured data series under different collection time interval

**Output:** predicted data series under different collection time interval

1.difp = dif(measured data)←**differential processing**

2. identify var = difp stationarity = (adf = 1) ←**stationarity test**

3.identify var = difp nlag = *p*+*d*+*q* outcov = weekday1←**white noise test**

4. identify var = difp nlag = *p*+*d*+*q* minic *p q*←**determining the model order**

5.estimate *p q* noint method = m1←**model parameter estimation**

6.forecast←**model prediction result**

### SDGM model

**Fig 1:**
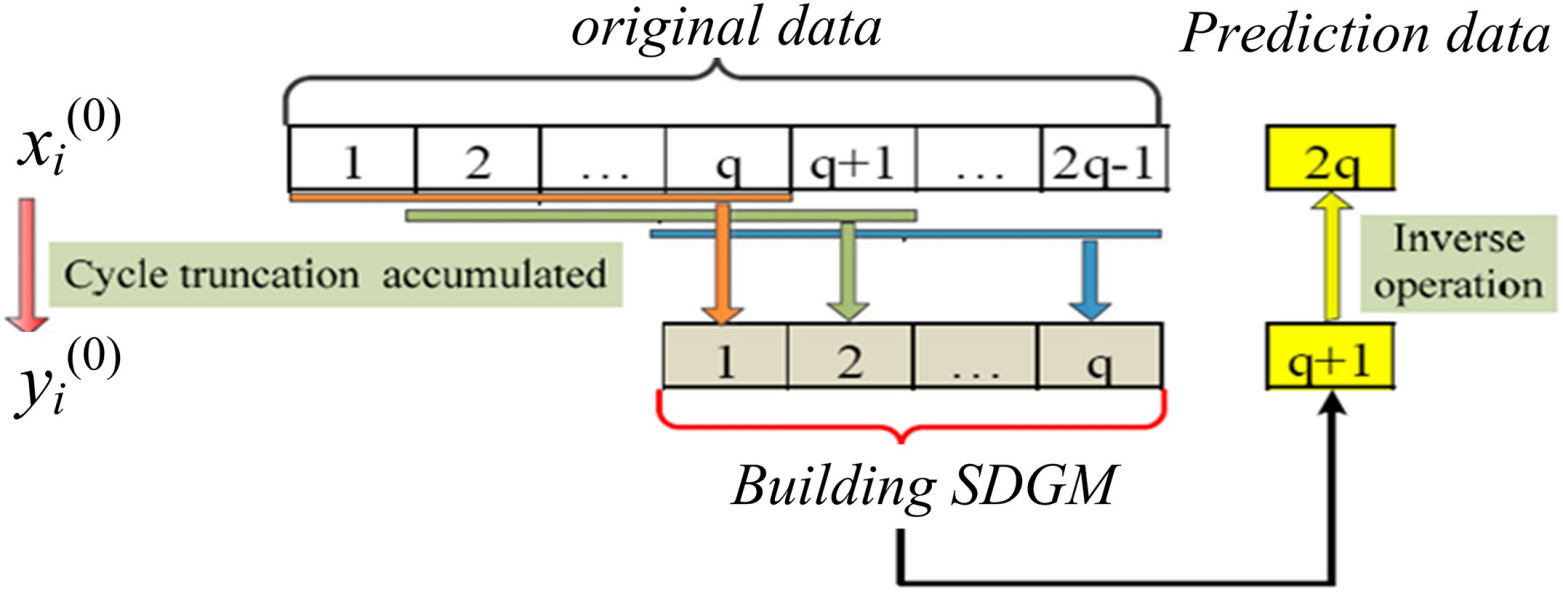
The process to obtain the CTAGO sequence.

The discrete grey model (DGM) is used to predict the cross-sectional data. However, if the original sequence is a seasonal sequence, the DGM is unable to capture the oscillation of the data, leading to poor prediction accuracy [55]. Therefore, the cycle truncation accumulated generating operation (CTAGO) is introduced as shown in Fig 1, The SDGM model which is an improved form of the DGM, considering the CTAGO operator is proposed.

Assume that *x*_*i*_^(0)^ is an original, seasonal sequence at cross-section *i*, *y*_*i*_^(0)^ represents the CTAGO sequence can be given by (2), *q* is periodic value, and mark *n*-*q*+1 is *r*.

$$\forall k\;=\;1,2,\cdots ,r;\;y_{i}^{\left(0\right)}(k)\;=\;CTAGO(x_{i}^{\left(0\right)}(k))\;=\;\sum_{j\;=\;1}^{q}x_{i}^{\left(0\right)}(k+j-1)$$ (2)

where *n* is total number of parameter; *q* is the periodic value; *k* is parameter number index; *i* is the cross-sectional position; $x_{i}^{\left(0\right)}(k)\;=\;{\left(x_{i}^{\left(0\right)}(1),x_{i}^{\left(0\right)}(2),\cdots ,x_{i}^{\left(0\right)}(r)\right)}^{T}$ is the original sequence; and $y_{i}^{\left(0\right)}(k)\;=\;{\left(y_{i}^{\left(0\right)}(1),y_{i}^{\left(0\right)}(2),\cdots ,y_{i}^{\left(0\right)}(r)\right)}^{T}$ is the CTAGO sequence.

The sequence *y*_*i*_^(1)^ can be calculated based on the first-order accumulated generating operation (1-AGO) as shown in the (3).

$$y_{i}^{\left(1\right)}(k)\;=\;\sum_{t\;=\;1}^{k}y_{i}^{\left(0\right)}(t),k\;=\;1,2,\cdots ,r$$ (3)

where *t* is parameter number index; and $y_{i}^{\left(1\right)}(k)\;=\;{\left(y_{i}^{\left(1\right)}(1),y_{i}^{\left(1\right)}(2),\cdots ,y_{i}^{\left(1\right)}(r)\right)}^{T}$ is the 1-AGO sequence of CTAGO sequence.

By combining (2) and (3), the following equation is obtained.

$$y_{i}^{\left(1\right)}(k)\;=\;\sum_{t\;=\;1}^{k}\sum_{j\;=\;1}^{q}x_{i}^{\left(0\right)}(t+j-1),k\;=\;1,2,\cdots ,r$$ (4)

In the above representation, Eq (3) defines the 1-AGO structure of CTAGO sequence. Eq (4) shows the relationship between 1-AGO sequence of CTAGO sequence *y*_*i*_^(1)^ and original sequence *x*_*i*_^(0)^. As shown in (3) and (4), the sequences *y*_*i*_^(1)^ is an ascending sequences. Therefore, Eq (5) is used to define the sequence increment relationship structure as follows.

$$y_{i}^{\left(1\right)}(k+1)\;=\;\beta_{1}y_{i}^{\left(1\right)}(k)+\beta_{2}$$ (5)

where *β*_1_ is the coefficient of least-squares estimation; and *β*_2_ is the coefficient of least-squares estimation.

The coefficients *β*_1_ and *β*_2_ can be estimated by (6) and (7).

$$\delta \;=\;B\hat{\beta }$$ (6)

$$\hat{\beta }\;=\;{\left[\beta_{1},\beta_{2}\right]}^{T}\;=\;{\left(B^{T}B\right)}^{-1}B^{T}\delta$$ (7)

where $\delta \;=\;{\left(y_{i}^{\left(1\right)}(2),y_{i}^{\left(1\right)}(3),\cdots ,y_{i}^{\left(1\right)}(r)\right)}^{T}$; and $B\;=\;[\begin{array}{cc} y_{i}^{\left(1\right)}(1) & 1\\ y_{i}^{\left(1\right)}(2) & 1\\ \vdots & \vdots \\ y_{i}^{\left(1\right)}(r-1) & 1 \end{array}]$.

The relationship between *y*_*i*_^(1)^ and original sequences *x*_*i*_^(0)^, can be calculated by (8) and (9).

$$\delta \;=\;C_{1}G_{1}X$$ (8)

$$B\;=\;C_{2}G_{2}M$$ (9)

where $C_{1}={\left[\begin{array}{ccccc} 1 & 1 & 0 & \ldots & 0\\ 1 & 1 & 1 & \ldots & 0\\ \vdots & \vdots & \vdots & \ddots & \vdots \\ 1 & 1 & 1 & \ldots & 1 \end{array}\right]}_{\left(r-1\right)\cdot r}$; $G_{1}={\left[\begin{array}{cccccccccc} 1 & 1 & \ldots & 1 & 0 & \ldots & 0 & 0 & \ldots & 0\\ 0 & 1 & \ldots & 1 & 1 & \ldots & 1 & 0 & \ldots & 0\\ \vdots & \vdots & \vdots & \vdots & \vdots & \ddots & \vdots & \vdots & \vdots & \vdots \\ 0 & 0 & 0 & 0 & 0 & \ldots & 1 & 1 & \ldots & 1 \end{array}\right]}_{r\cdot n}$; $C_{2}={\left[\begin{array}{ccccc} 1 & 0 & 0 & \ldots & 0\\ 1 & 1 & 0 & \ldots & 0\\ \vdots & \vdots & \vdots & \ddots & \vdots \\ 1 & 1 & 1 & \ldots & 1 \end{array}\right]}_{\left(r-1\right)}$; $G_{2}={\left[\begin{array}{cccccccccc} 1 & 1 & \ldots & 1 & 0 & \ldots & 0 & 0 & \ldots & 0\\ 0 & 1 & \ldots & 1 & 1 & \ldots & 0 & 0 & \ldots & 0\\ \vdots & \vdots & \vdots & \vdots & \vdots & \ddots & \vdots & \vdots & \vdots & \vdots \\ 0 & 0 & 0 & 0 & 0 & \ldots & 1 & 1 & \ldots & 1 \end{array}\right]}_{\left(r-1\right)\cdot \left(n-1\right)}$; $M=\left[\begin{array}{cc} x_{i}^{\left(0\right)}\left(1\right) & 1\\ x_{i}^{\left(0\right)}\left(1\right) & 0\\ \vdots & \vdots \\ x_{i}^{\left(0\right)}\left(n-1\right) & 0 \end{array}\right]$; and $X={\left(x_{i}^{\left(0\right)}\left(1\right),x_{i}^{\left(0\right)}\left(2\right),\cdots ,x_{i}^{\left(0\right)}\left(n-1\right)\right)}^{T}$.

By combining (7) to (9), the solving process of coefficients *β*_1_ and *β*_2_ can be converted into the (10).

$$\hat{\beta }\;=\;({{\left(C_{2}G_{2}M\right)}^{T}C_{2}G_{2}M)}^{-1}{\left(C_{2}G_{2}M\right)}^{T}C_{1}G_{1}X$$ (10)

The solution of SDGM is proposed by the (11). The time response structure of CTAGO sequence can be presented by (12). Eq (13) defines the solution of the corresponding seasonal original sequence *x*_*i*_^(0)^ after the inverse operation.

$${\hat{y}}_{i}^{\left(1\right)}(t+1)\;=\;{{(y_{i}^{\left(0\right)}(1)-\frac{\beta_{2}}{1-\beta_{1}})\beta }_{1}}^{t}+\frac{\beta_{2}}{1-\beta_{1}}$$ (11)

$${\hat{y}}_{i}^{\left(0\right)}(t+1)\;=\;y_{i}^{\left(1\right)}(t+1)-y_{i}^{\left(1\right)}(t)\;=\;(\beta_{1}-1)(y_{i}^{\left(0\right)}(1)-\frac{\beta_{2}}{1-\beta_{1}})\beta_{1}^{t-1}$$ (12)

∀t=q,q+1,⋯,n

$${\hat{x}}_{i}^{\left(0\right)}(t+1)\;=\;y_{i}^{\left(0\right)}(t-q+2)-y_{i}^{\left(0\right)}(t-q+1)+x_{i}^{\left(0\right)}(t-q+1)$$ (13)

where ${\hat{x}}_{i}^{\left(0\right)}(t)$ is the original sequence predicted by using SDGM;$\;{\hat{y}}_{i}^{\left(0\right)}(t)$ is the CTAGO sequence predicted by using SDGM; and ${\hat{y}}_{i}^{\left(1\right)}(t)$ is the I-AGO sequence of CTAGO sequence predicted by using SDGM.

The SDGM algorithm in SAS is shown in Algorithm 2.

**Algorithm 2: SDGM**

**Input:** measured data series under different collection time interval

**Output:** predicted data series under different collection time interval

1.input data a0 (t id xt) ←**input measured data series a0**

2. by id t;

 x1 = lag(xt) x2 = lag2(xt) … … x6 = lag6(xt);

 y = sum(xt,x1,x2,x3,x4,x5,x6)

 set a1←**seasonal processed and stored in a1**

3.use a1

 yt+y; index = 1; zt = -(yt+LAG(yt)/2);

 set a2←**accumulated a1 and stored in a2**

4.use a2

 proc iml

 read all var{zt index} into B

 read all var{y} into yn

 ahat = inv(B′*B)*B′*yn; ahatt = ahat′;na = {a u}

 creat a3 from ahatt [colname = na]; ←**IML module process**

5.use a3

 yt1 = (xt0-u/a)*exp(-a*(t-1))+u/a;

 yt0 = (xt0-u/a)*exp(-a*(t-2))+u/a;

 xp = yt1-yt0;

 set a4 ←**output the prediction results a4**

### SARIMA-SDGM hybrid model

In this study, a SARIMA-SDGM hybrid model was proposed for short-term traffic speed prediction. In practical, SARIMA model is used to forecast the periodic time series data. SDGM(1,1) is used to forecast the cross-sectional data that has weekly seasonal characteristics. The structure of hybrid model is given in (14).

$$V_{t+1}\;=\;w_{t}^{sarima}V_{t+1}^{sarima}+w_{t}^{sdgm}V_{t+1}^{sdgm}$$ (14)

where *t* is the time index; *V*_*t*_ is the predicted value by using hybrid model; $V_{t}^{sarima}\;$ is the predicted value by using SARIMA; $V_{t}^{sdgm}$ is the predicted value by using SDGM; $w_{t}^{sarima}\;$ is the weighted value by using SARIMA; and $w_{t}^{sdgm}$ is the weighted value by using SDGM.

The weight in the hybrid model is determined by the performance of the single model prediction at time *t*. The lower nearness degree between the actual value and predicted value is, the smaller the weight is. The weight algorithm of hybrid prediction model is as follows.

**Step 1**, Estimating the prediction value by SARIMA model and SDGM model as given in (15) and (16), respectively.

{Vtsarima(k)=(Vtsarima(1),⋯,Vtsarima(r))V^tsarima(k)=(V^tsarima(1),⋯,V^tsarima(r)) (15)

{Vtsdgm(k)=(Vtsdgm(1),⋯,Vtsdgm(r))V^tsdgm(k)=(V^tsdgm(1),⋯,V^tsdgm(r)) (16)

where ${\hat{V}}_{t}^{sarima}(k)\;$ is the original data sequence by using SARIMA; $V_{t}^{sarima}(k)\;$ is the predicted data sequence by using SARIMA; ${\hat{V}}_{t}^{sdgm}(k)\;$ is the original data sequence by using SDGM; and $V_{t}^{sdgm}(k)$ is the predicted data sequence by using SDGM.

**Step 2**, Calculating the corresponding nearness degree $\rho_{t}^{sarima}$ and $\rho_{t}^{sdgm}$ as given in (17).

{ρtsarima=1/(1+|Vtsarima-V^tsarima|)ρtsdgm=1/(1+|Vtsdgm-V^tsdgm|) (17)

where $\rho_{t}^{sarima}\;$ is the nearness degree by using SARIMA; and ${\;\rho }_{t}^{sdgm}$ is the nearness degree by using SDGM.

**Step 3**, Determining the corresponding weighted coefficients by the nearness degree as given in (18).

{wtsarima=ρtsarima/(ρtsarima+ρtsdgm)wtsdgm=ρtsdgm/(ρtsarima+ρtsdgm) (18)

where $w_{t}^{sarima}$ is the weighted value by using SARIMA; and $w_{t}^{sdgm}$ is the weighted value by using SDGM.

### Machine learning methods

Two machine learning methods, including ANN model and SVR model, were introduced for comparison.

ANN is a data-driven model and has the capability of complex mapping between inputs and outputs that enables appropriating nonlinear functions [56]. The basic structure of ANN model consists of multiple layers, including one input layer, one output layer, and one or more hidden layers. Each layer comprises several nodes connected to the nodes in neighboring layers. With the application of ANN model, the inputs can be previous lagged traffic speed values while the outputs can provide future traffic speed forecasts. The input-output relation of neural network models for prediction can be represented as follows

$$\hat{v}(t+d)\;=\;F(v(t),v(t-1),\cdots ,v(t-n))$$ (19)

where *v(t)* presents the traffic speed at the time *t*; and $\hat{v}(t+d)$ is the predicted traffic speed at the time *t+d*; *F(·)* is a nonlinear function; *d* is the collection time interval of traffic speed data.

SVR is a regression analysis model based on the support vector machine (SVM) [57]. The model is to map the input data into a higher dimensional feature space through a nonlinear mapping, and then a linear regression problem is obtained and solved in this feature space. The goal of SVR model is to find a function *f*(*x*_*i*_) that has at most *ε* deviation from the actually obtained targets *y*_*i*_ for all the training data. SVR model neglects the errors that are less than *ε*, and the loss will be calculated when the absolute value of the error between *f*(*x*_*i*_) and *y*_*i*_ is larger than *ε*. The structure of SVR model can be represented as follows

$$\underset{w,b}{\text{min}}\;\frac{1}{2}{\left\|w\right\|}^{2}+C\sum_{i\;=\;1}^{m}l_{\epsilon }(f(x_{i})-y_{i})$$ (20)

where *ℓ*_ϵ_ presents the *ϵ*-insensitive loss function; *C* is the constant; *w* can be completely described as a linear combination of the training patterns *x*_*i*_; *b* turns out to be the coefficient of the optimization process.

### Model performance measures

The performance of SARIMA-SDGM model was compared with that of SARIMA model, SDGM model, ANN model, and SVR model. As well, the prediction results of SARIMA-SDGM models under different data collection time were compared. Three indicators including the mean absolute error (MAE), mean absolute percentage error (MAPE), and the root mean square error (RMSE) were used for the comparison. The following equations are given as:

$$MAE\;=\;\frac{1}{n}\sum_{i\;=\;1}^{n}\left|X_{i}-{\hat{X}}_{i}\right|$$ (21)

$$MAPE\;=\;\frac{100}{n}\sum_{i\;=\;1}^{n}\left|\frac{X_{i}-{\hat{X}}_{i}}{X_{i}}\right|$$ (22)

$$RMSE\;=\;\sqrt{\frac{1}{n}\sum_{i\;=\;1}^{n}{\left(X_{i}-{\hat{X}}_{i}\right)}^{2}}$$ (23)

where *n* is the total number of observations; *X*_*i*_ is the predicted parameter value; and ${\hat{X}}_{i}\;$ is the original parameter value.

## Data preparation

### Study location

**Fig 2:**
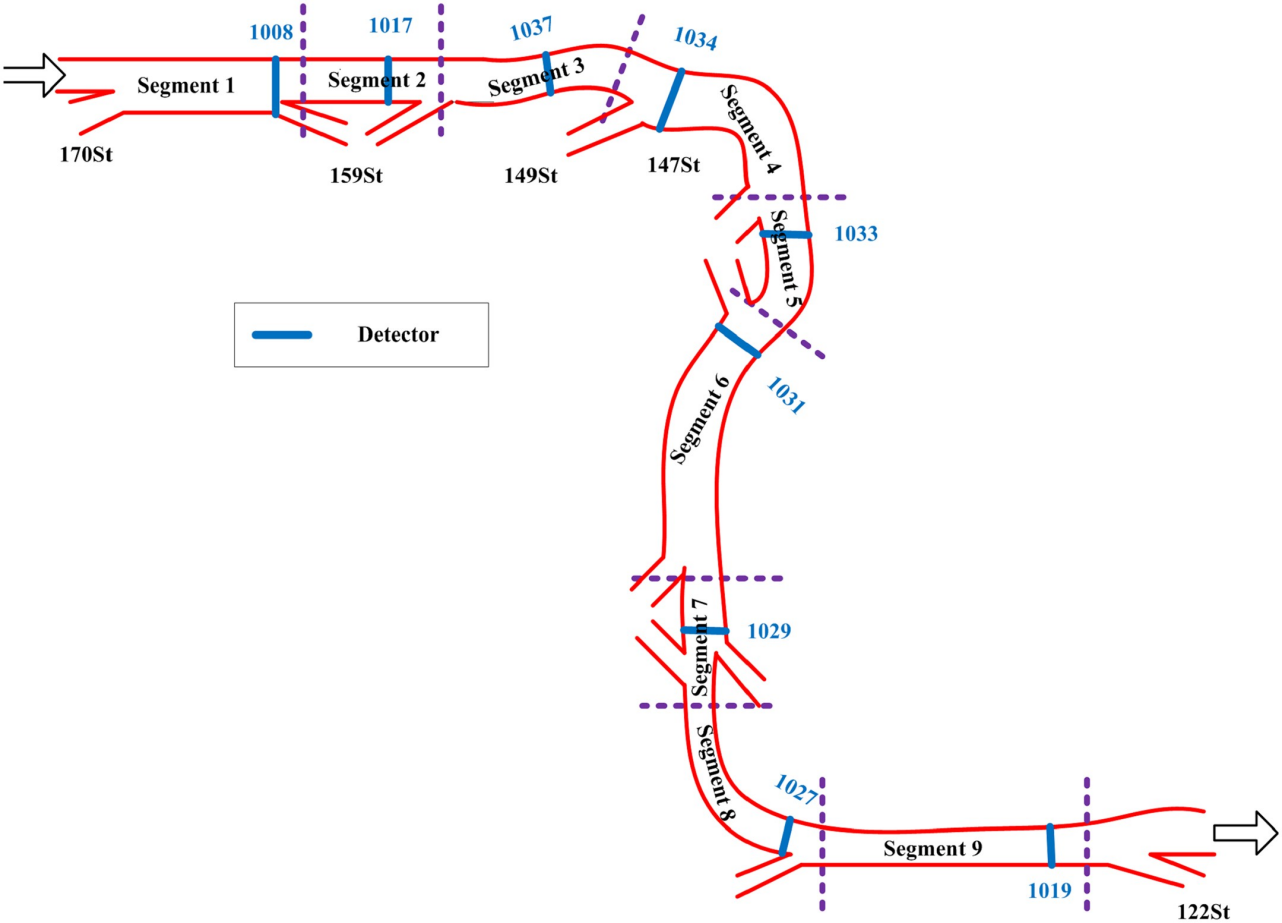
Study segmentation of freeway.

Traffic speed data was collected from an urban freeway corridor that called Whitemud Drive in Edmonton, Canada, through the vehicle detection stations (VDS, including loop detector and traffic video camera). The west to east direction segment between 170th street to 122th street was selected in this study. For this study, the selected segment was divided into nine segments based on the detectors location. Each segment is approximately 800 m. Fig 2 shows the selected freeway and the nine segments (http://www.openits.cn/openData1/700.jhtml).

### Data collection

Traffic speed data was available from online open data [58]. Twenty-four days (5 August to 28 August 2015) of speed data was extracted from the VDS system in the open data [58]. These data were selected to test the model performance. Table 1 shows the speed data collection time and location. To compare the prediction performance under different traffic speed data collection time interval test, the original speed data is aggregated into 11 data collection time intervals (1 min, 3 min, 5 min, 8 min, 10 min, 12 min, 15 min, 18 min, 20 min, 25 min, and 30 min) for each segment as shown in Table 2.

**Table 1:** Data collection time and location.

Segment ID	VDS	Region	Freeway	Numbers of lanes	Start	End	AM (Time)	PM (Time)
1	1008	Edmonton	Whitemud Drive	4	5/8/2015	28/8/2015	7–9	5–7
2	1017	Edmonton	Whitemud Drive	3	5/8/2015	28/8/2015	7–9	5–7
3	1037	Edmonton	Whitemud Drive	3	5/8/2015	28/8/2015	7–9	5–7
4	1034	Edmonton	Whitemud Drive	4	5/8/2015	28/8/2015	7–9	5–7
5	1033	Edmonton	Whitemud Drive	3	5/8/2015	28/8/2015	7–9	5–7
6	1031	Edmonton	Whitemud Drive	4	5/8/2015	28/8/2015	7–9	5–7
7	1029	Edmonton	Whitemud Drive	3	5/8/2015	28/8/2015	7–9	5–7
8	1027	Edmonton	Whitemud Drive	3	5/8/2015	28/8/2015	7–9	5–7
9	1019	Edmonton	Whitemud Drive	3	5/8/2015	28/8/2015	7–9	5–7

Note: AM: morning; PM: afternoon.

**Table 2:** Groups by different collection time interval.

Group	Time interval	Samples	Mean (km/h)	Max (km/h)	Min (km/h)	Std.
G1	1 min	51840	87.12	110.25	61.5	6.17
G2	3 min	17280	87.15	103.92	69	5.81
G3	5 min	10368	87.53	103.5	71.9	5.69
G4	8 min	6912	87.16	103.44	74.47	5.54
G5	10 min	5184	87.19	102.2	74.58	5.51
G6	12 min	4320	87.85	101.60	74.35	5.49
G7	15 min	3456	87.17	100.63	75.17	5.44
G8	18 min	2850	87.19	100.03	74.42	5.41
G9	20 min	2592	87.13	100.30	75.89	5.40
G10	25 min	2074	87.22	99.23	76.23	5.38
G11	30 min	1728	87.20	99.11	76.32	5.38

## Results and analysis

### Model performance comparison

**Fig 3:**
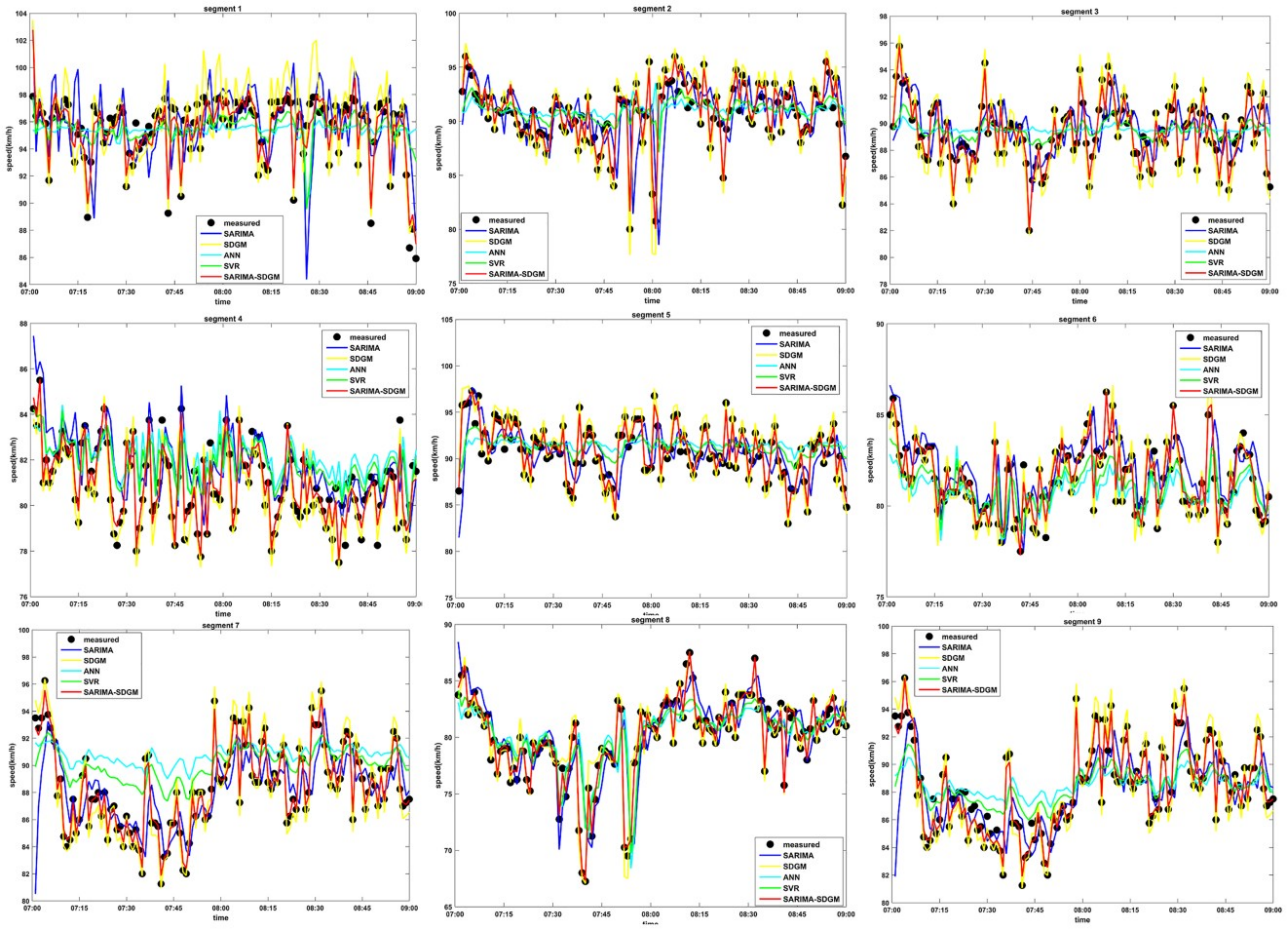
1min-Speed prediction by using different models for AM, August 28, 2015.

**Fig 4:**
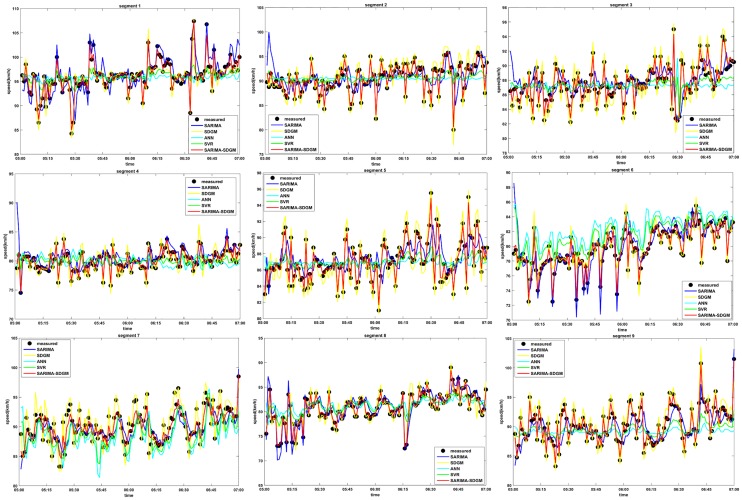
1min-Speed prediction by using different models for PM, August 28, 2015.

In order to investigate the performance of the proposed SARIMA-SDGM model, the prediction results of the five candidate models were compared. The speed data which was aggregated into 1 min was utilized for the models’ performance comparison. Fig 3 shows the measured speed and the predicted speed of different models for the nine segments in the morning peak hours. As well, Fig 4 shows the measured speed and the predicted speed of different models for the nine segments in the afternoon peak hours. The figures show that the predicted speed of the SARIMA-SDGM model is closer to the field-measure speed compared to that of SARIMA model, SDGM model, ANN model, and SVR model. This finding indicates that the SARIMA-SDGM model can better capture the variation characteristics of the filed-measured speed.

To further quantitative measure the predictive accuracy of the models, the model performance measures were also shown in Tables 3 and 4. As shown in Tables 3 and 4, the SARIMA-SDGM model performs best with the lowest MAE, MAPE and RMSE, indicating that accounting for the characteristics of the traffic speed sequence over time correlation and spatial correlation could significantly improve the prediction results. However, the SARIMA model shows the least performance among the five developed models, which is expected since this model has poor response to sudden changes of speed trend. The performances of ANN model and SVR model are between that of SARIMA-SDGM model and SARIMA model. Moreover, the SVR model has a better performance than the ANN model. The performance of SDGM model are compared to that of ANN model and SVR model, indicating that by converting the volatility sequence traffic speed sequence into a stable sequence through the 1-AGO method could improve the prediction results. T.

**Table 3:** Predictive accuracy performance for different segment for AM.

Segment ID	MAE					MAPE					RMSE				
	(A)	(B)	(C)	(D)	(E)	(A)	(B)	(C)	(D)	(E)	(A)	(B)	(C)	(D)	(E)
1	3.38	2.75	2.54	3.08	2.81	3.32%	2.79%	2.55%	3.31%	2.94%	4.36	3.95	3.58	4.24	4.11
2	3.24	2.91	2.80	2.90	2.87	3.44%	2.99%	2.88%	2.98%	2.92%	4.53	3.69	3.62	4.21	4.09
3	3.02	2.52	2.25	2.84	2.53	3.16%	2.75%	2.41%	2.87%	2.55%	3.24	2.87	2.68	2.32	2.23
4	2.76	2.49	2.36	2.61	2.44	3.49%	2.63%	2.44%	2.97%	2.59%	3.21	2.66	2.51	2.92	2.76
5	2.44	2.27	1.61	2.49	2.31	2.97%	2.48%	2.37%	2.75%	2.51%	3.06	2.94	2.08	3.21	2.91
6	2.73	2.35	1.91	2.36	2.17	3.13%	2.55%	2.22%	5.66%	3.94%	3.44	2.77	2.59	4.81	3.50
7	3.22	2.54	2.38	3.08	2.53	3.28%	2.66%	2.42%	2.55%	2.47%	3.13	2.87	2.45	2.65	2.58
8	4.53	3.88	3.45	2.38	2.32	4.19%	3.74%	3.55%	3.11%	2.92%	4.74	3.92	3.76	3.50	2.97
9	3.10	2.50	2.11	2.23	2.13	3.34%	2.74%	2.46%	2.52%	2.48%	3.18	2.75	2.45	2.73	2.68
Average	3.16	2.69	2.38	2.66	2.46	3.37%	2.81%	2.59%	3.19%	2.81%	3.65	3.16	2.86	3.40	3.09

Note: predictive accuracy performance for different segments: (A) SARIMA model, (B) SDGM model, (C) SARIMA-SDGM model, (D) ANN model, (E) SVR model

**Table 4:** Predictive accuracy performance for different segment for PM.

Segment ID	MAE					MAPE					RMSE				
	(A)	(B)	(C)	(D)	(E)	(A)	(B)	(C)	(D)	(E)	(A)	(B)	(C)	(D)	(E)
1	4.34	3.85	3.69	3.55	3.34	4.54%	3.84%	3.51%	3.65%	3.42%	5.64	5.12	4.93	5.50	5.20
2	4.23	3.74	3.59	3.69	3.62	3.78%	3.15%	3.03%	3.83%	3.74%	4.31	3.75	3.57	4.03	3.94
3	3.32	2.86	2.69	3.12	3.03	3.23%	2.98%	2.79%	3.48%	3.25%	3.54	3.22	3.07	3.79	3.66
4	3.44	3.00	2.78	3.71	3.37	3.65%	3.25%	2.99%	3.98%	3.31%	3.11	2.95	2.81	4.14	3.63
5	3.95	3.21	2.98	3.22	3.08	3.78%	3.35%	3.13%	3.39%	3.15%	3.48	3.12	3.01	3.54	3.11
6	3.12	2.66	2.57	2.91	2.68	3.21%	2.94%	2.72%	3.14%	2.96%	3.08	2.88	2.71	3.36	3.33
7	3.81	3.21	3.07	3.98	3.68	3.91%	3.33%	3.15%	4.28%	4.04%	4.27	3.72	3.50	4.35	4.17
8	2.89	2.35	2.29	2.52	2.37	3.05%	2.84%	2.54%	3.17%	2.81%	3.14	2.65	2.40	3.28	2.86
9	3.35	2.95	2.67	3.33	3.13	3.30%	3.11%	2.75%	3.54%	3.40%	3.65	3.22	3.00	3.55	3.45
Average	3.61	3.09	2.93	3.34	3.14	3.61%	3.20%	2.96%	3.61%	3.34%	3.80	3.40	3.22	3.95	3.71

Note: predictive accuracy performance for different segments: (A) SARIMA model, (B) SDGM model, (C) SARIMA-SDGM model, (D) ANN model, (E) SVR model

As shown in Table 3, the SARIMA-SDGM model could improve the average prediction accuracy by 32.7%, 30.1%, and 27.9% respectively compared with the SARIMA model according to the MAE, MAPE, and RMSE measures. In addition, SDGM model could improve the average prediction accuracy by 17.4% and 15.7% compared with the SARIMA model according to the MAE and RMSE measures. This result is consistent with several previous studies [40, 50] which showed that the SDGM model outperformed the SARIMA model. The similar results can be also found for the afternoon peak hours traffic speed prediction results in Table 4.

### Model performance under different time intervals

**Fig 5:**
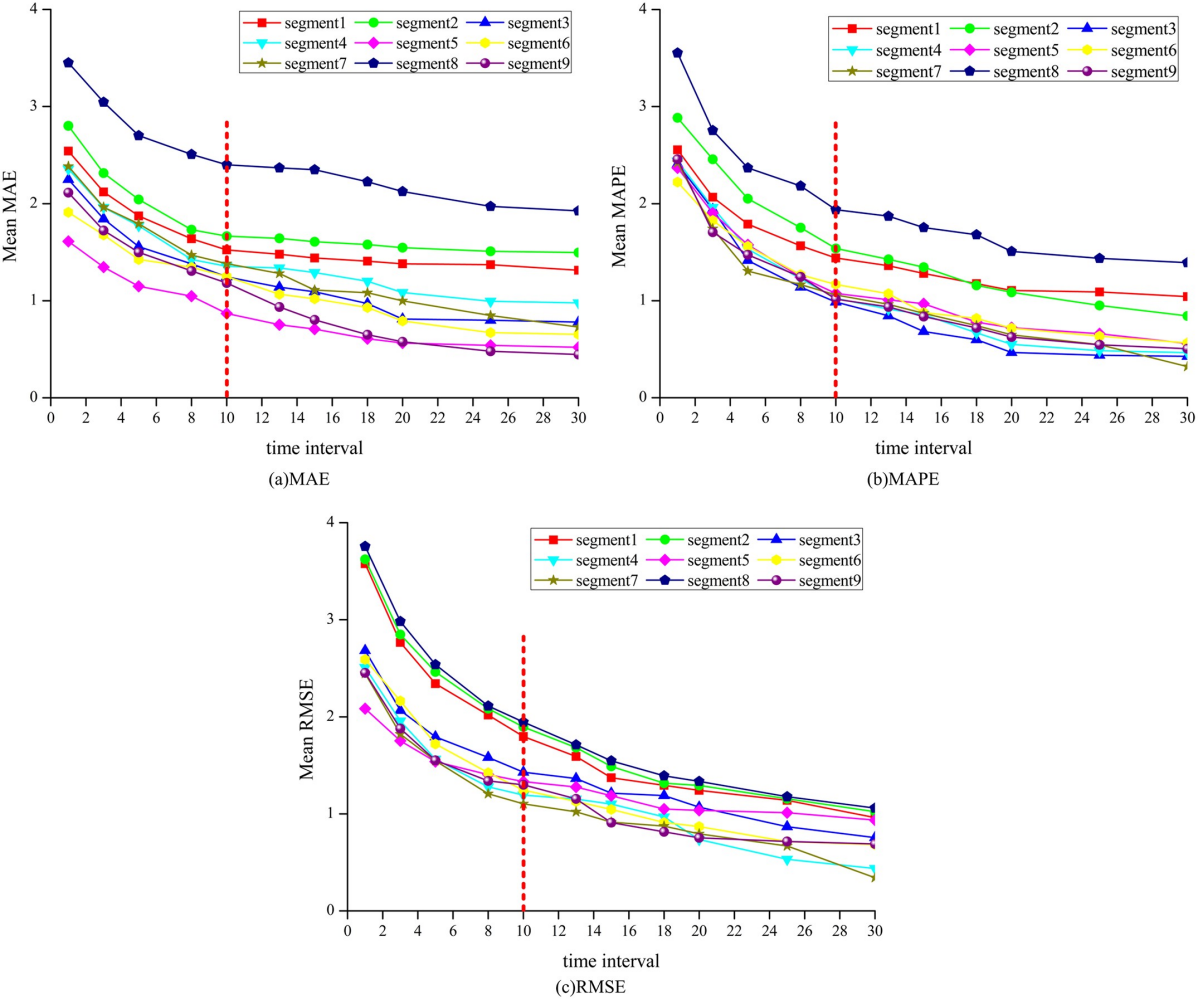
Predictive accuracy performance for AM.

**Fig 6:**
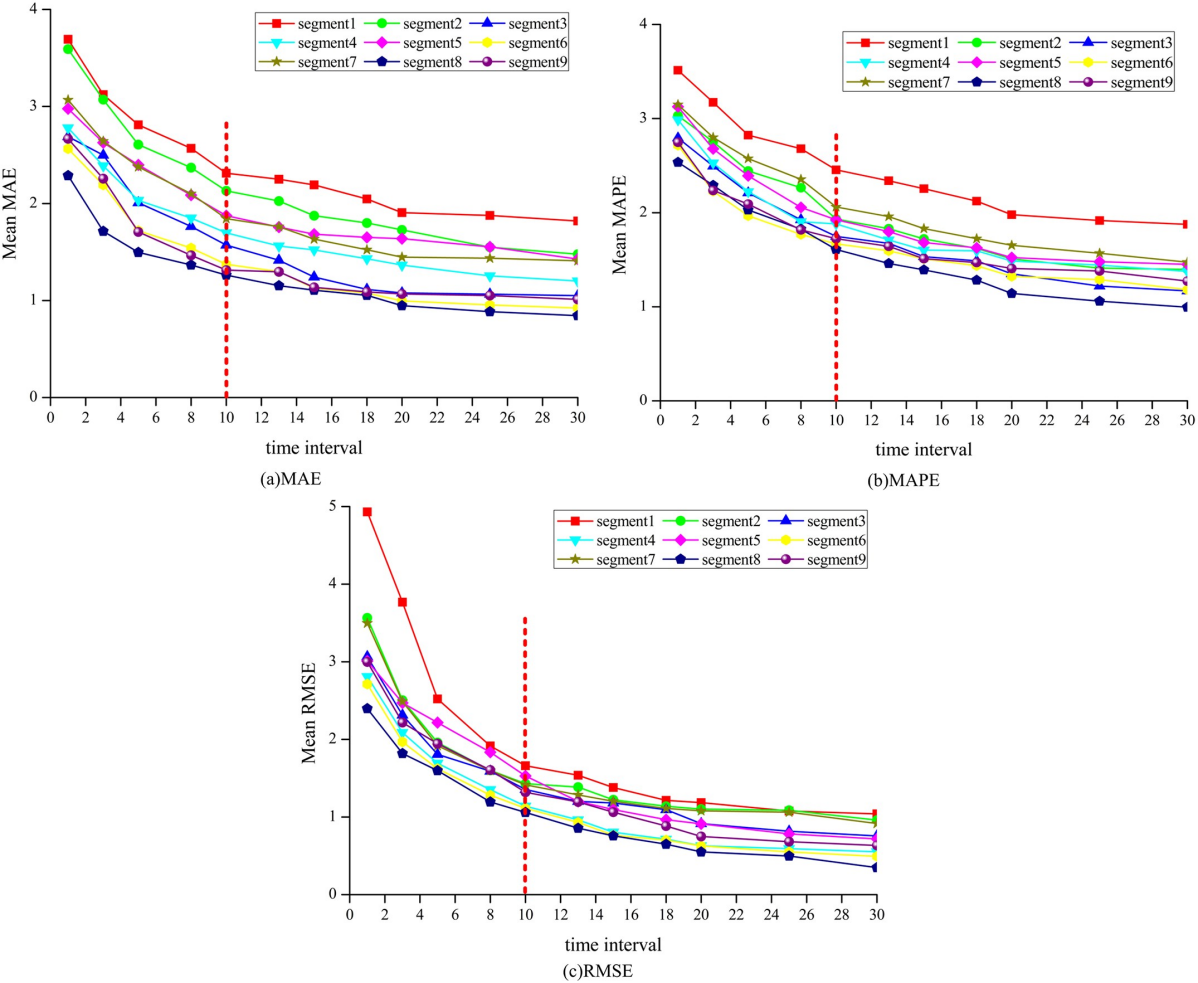
Predictive accuracy performance for PM.

To investigate the impact of data collection time interval on the traffic speed prediction accuracy, SARIMA-SDGM model was used to predict the traffic speed under different time intervals (i.e. 1 min, 3 min, 5 min, 8 min, 10 min, 12 min, 15 min, 18 min, 20 min, 25 min, and 30 min). Figs 5 and 6 show similar trends for all the segments across the measures under different time interval during morning and afternoon peak hours. As shown in Figs 5 and 6, the prediction accuracy improves with the increase in time interval. For example, the average MAE, MAPE, and RMSE are approximately 2.65, 2.80% and 3.10 respectively for all segments at the time interval of 1-min. By comparison, the average MAE, MAPE, and RMSE are 1.35, 1.30% and 1.20 respectively for all segment at time interval of 10-min. The decrease in these three indicators indicates the improvement of the speed prediction. This finding meet the fact that the increase of the data collection time interval can reduce the volatility of traffic speed, thereby making the speed series more stable and thus more predictable [11]. The observed association of increased prediction accuracy with increased data collection time interval is consistent with that from other valid forecasting methods [59].

Moreover, as shown in Figs 5 and 6, the lines between time intervals 1 min and 10 min show a sharp decrease trend for all the segments. Whereas, the lines between time intervals 10 min and 30 min show a relatively flat pattern. This finding indicates that the traffic speed prediction results can be improved significantly with the increase in time interval when the time interval is smaller than 10 min. In addition, the prediction results yield stable prediction accuracy when the time interval is greater than 10 min. This finding can be explained with the stability of the speed data under different time interval. The standard deviation of traffic speed is approximately 5.40 when the time interval is greater than 10 min, while the standard deviation is approximately 6.00 when the time interval is smaller than 10 min. The result indicates that the accurate prediction of traffic speed could be generated using 10 min and longer time interval based on the SARIMA-SDGM model structure.

## Discussion and conclusion

This study investigated the impact of data collection time interval on short-term traffic speed prediction. A SARIMA-SDGM model was proposed for predicting the traffic speed under different data collected time interval. Speed data were collected from an urban freeway in Edmonton, Canada. The parametric model (SARIMA model and SDGM model) and nonparametric model (ANN model and SVR model) were also developed and compared with SARIMA-SDGM model using three model performance measures. The model performance under different time interval was compared to provide insights into the effects of data collection time interval.

The results showed that the SARIMA-SDGM model performed best with the lowest MAE, MAPE and RMSE. Whereas, the SARIMA model showed the least performance among the five developed models. The results indicated that SARIMA-SDGM model can better capture the variation characteristics of the filed-measured traffic speed data. For the model performance under different data collection time interval, the results showed that the five model performance measures decreased with the increase in time interval. The results indicated that the prediction accuracy improves with the increase in time interval. Moreover, the SARIMA-SDGM model can yield stable prediction accuracy for traffic speed data with greater than 10 min data collection time intervals.

There are some limitations to this study. (a)This study utilized the traffic speed data from 9 segments. The connection between adjacent segments may affect the traffic speed prediction performance. Future work should investigate relationship of traffic speed between the adjacent segments. (b) Uncertainty of traffic speed prediction was considered as an inevitable problem due to the stochastic volatility feature. Uncertainty model and uncertainty quantification analysis can be applied to these speed data series for short-term prediction. (c) This study shown that SARIMA-SDGM model can yield the better prediction results, but still cannot be applied in the real time traffic speed prediction. Thus, the online algorithm for short-term traffic speed prediction using state-of-the-arts methods such as Kalman filters was also a valuable research.
